# Associations between measures of physical fitness and cognitive performance in preschool children

**DOI:** 10.1186/s13102-022-00470-w

**Published:** 2022-05-01

**Authors:** Kristin Wick, Susi Kriemler, Urs Granacher

**Affiliations:** 1 University of Applied Sciences for Sports and Management Potsdam, Am Luftschiffhafen 1, 14471 Potsdam, Germany; 2 Division of Training and Movement Sciences, University of Potsdam, Potsdam, Germany; 3 Epidemiology, Biostatistics and Prevention Institute, University of Zurich, Zurich, Switzerland

**Keywords:** Motor skills, Cognitive skills, Attention, Kindergarten

## Abstract

**Background:**

Given that recent studies report negative secular declines in physical fitness, associations between fitness and cognition in childhood are strongly discussed. The preschool age is characterized by high neuroplasticity which effects motor skill learning, physical fitness, and cognitive development. The aim of this study was to assess the relation of physical fitness and attention (including its individual dimensions (quantitative, qualitative)) as one domain of cognitive performance in preschool children. We hypothesized that fitness components which need precise coordination compared to simple fitness components are stronger related to attention.

**Methods:**

Physical fitness components like static balance (i.e., single-leg stance), muscle strength (i.e., handgrip strength), muscle power (i.e., standing long jump), and coordination (i.e., hopping on one leg) were assessed in 61 healthy children (mean age 4.5 ± 0.6 years; girls n = 30). Attention was measured with the “Konzentrations-Handlungsverfahren für Vorschulkinder” [concentration-action procedure for preschoolers]). Analyses were adjusted for age, body height, and body mass.

**Results:**

Results from single linear regression analysis revealed a significant (*p* < 0.05) association between physical fitness (composite score) and attention (composite score) (standardized ß = 0.40), showing a small to medium effect (F^2^ = 0.14). Further, coordination had a significant relation with the composite score and the quantitative dimension of attention (standardized ß = 0.35; *p* < 0.01; standardized ß =  − 0.33; *p* < 0.05). Coordination explained about 11% (composite score) and 9% (quantitative dimension) of the variance in the stepwise multiple regression model.

**Conclusion:**

The results indicate that performance in physical fitness, particularly coordination, is related to attention in preschool children. Thus, high performance in complex fitness components (i.e., hopping on one leg) tends to predict attention in preschool children. Further longitudinal studies should focus on the effectiveness of physical activity programs implementing coordination and complex exercises at preschool age to examine cause-effect relationships between physical fitness and attention precisely.

## Background

An increasing number of children suffer from the so-called exercise deficit disorder which is a condition characterized by reduced levels of physical activity that are below current recommendations of at least 60 min daily moderate-to-vigorous physical activity [1]. As a consequence, researchers have reported low levels of physical fitness and even tendencies of negative secular declines in physical fitness, particularly for cardiorespiratory endurance (especially between the years 1981 to 2000) [2] and muscle strength and power, in school-aged children [3, 4]. Thereby, the amount of declines in physical fitness reported within the mentioned reviews varied between countries [2–4]. Given that physical activity and fitness are rather robust phenomena that track from childhood to adulthood [5], it is important to promote physical activity and fitness at an early age to enable a healthy upbringing. Longitudinal studies [6] underline the positive effects of physical activity and physical fitness by showing that regular performance of aerobic exercise and perceptional motor training (3/week) over 36 weeks (academic school year) has the potential to improve not only cardiorespiratory endurance (i.e., aerobic fitness) but also cognitive (e.g., intelligence quotient) and academic performance (e.g., math and reading achievement) in school-aged children. Wick and colleagues [7] demonstrated that even in preschool children a 10-weeks (3/week) integrative strength-dominated exercise program was effective in significantly increasing physical fitness (i.e., standing long jump) and cognitive performance (i.e., attention). Moreover, cross-sectional studies in preschool children confirmed these findings. Greier and Drenowatz [8] showed weak correlations between balancing backwards (r = 0.21), jumping to and fro sideways (r = 0.24), and visuospatial working (measured with the “Human-Drawing-Test”) in children 5–6 years old. Similarly, Voelcker-Rehage [9] reported significant moderate relations between reaction time (r = 0.41), coordination (r = 0.30), and fine motor skills (r = 0.34) and cognitive performance (i.e., visual processing) in 4- to 6-year-old children. Niederer and colleagues [10] were focusing only on a sum score of attention exploring weak correlations between measures of aerobic fitness (r = 0.25), agility (r =  − 0.11), and attention in 245 preschool children. Accordingly, a systematic review in 4–16 year old children found that fine motor skills, bilateral body coordination, and speed of movement (e.g., foot tapping, running in a zigzag) had moderate to strong correlations with cognitive performance (i.e., memory, visual processing, executive functions, fluid intelligence) compared to gross motor and object control skills [11]. These findings demonstrate that especially in early childhood different domains of physical fitness, particularly coordination [8, 10, 11], seem to be related to cognitive performance (i.e., attention, memory, visual processing).

The reported associations between physical fitness and cognitive performance as well as the impact of physical exercise programs on cognitive performance are most likely caused by increased brain oxygenation based on an increased blood flow [12]. In addition, an increased neurotransmitter concentration which encourage information processing and an enhanced growth factor concentration which stimulate brain plasticity and neuronal cell connectivity are possible explanations how physical activity and exercise may effect cognitive performance [13, 14]. Thereby, the preschool age plays a decisive role during maturation. First, early childhood compared to late childhood and adolescence is characterized by accelerated cognitive maturation and rapid cognitive developmental trajectories [15]. Second, the acquisition and mastery of fundamental movement skills predominantly evolve during the preschool years [16] emphasizing the close relationship between motor and cognitive development during early age [17]. Fundamental movement skills build the foundation to achieving and maintaining physical fitness [18]. Both parameters mutually influence one other [19] and are essential parts for a continuously active lifestyle. In the context of this study, physical fitness is defined as the ability to carry out daily tasks with alertness, vigor, and sufficient energy [20]. Third, possible deficits in motor or cognitive development that may negatively influence following developmental stages [2–4] may be detected as early as possible.

Accordingly, the aim of this study was to assess the relationships of physical fitness with attention and its individual dimensions (quantitative, qualitative). Further, we aimed on finding physical fitness components (i.e., static balance, muscle strength, power, and coordination) that predict the variance of attention in a convenience sample of healthy preschool children. Cognitive performance comprises a whole set of mental actions and processes that contribute to perception, memory, attention, and intellect [21]. In our study we are concentrating on attention as it has an impact on school readiness [22], positively influence the transition from preschool to primary school and predict academic achievement in the long term during the school years [23, 24]. Moreover, attention composes a quantitative (working speed) and a qualitative (working accuracy) dimension which characterize levels of attentional capacity. Given that preliminary research has shown better cognitive performance in physically fit children [6, 8, 10], we further hypothesized that fitness components which need precise coordination during execution (e.g., hopping on one leg) and require higher order cognitive skills are stronger related to attention compared to fitness components which are more fundamental constructs without the need of a high skill level (i.e., handgrip strength) [11].

## Methods

An exploratory study design was used to examine physical fitness and cognitive performance in preschoolers from a convenience sample of three kindergartens located in eastern regions of Germany [7]. Sixty-one children (boys n = 31; girls n = 30) with a mean age of 4.5 ± 0.6 years and a range of 4–6 years (i.e., 42–74 months, 58.7 ± 7.3 months) participated in the study, which was approved by the local ethics research committee (submission No. 34/2018). Additionally, the study was conducted in accordance with the latest version of the Declaration of Helsinki. Prior to the start of the study, parents or legal representatives received written information on the aims of the study and the study design including potential risks and benefits. Parents or legal representatives of all participating children provided their written informed consent before the study started. An a priori power analysis was computed using G × Power (Version 3.1.9.2, University of Kiel, Kiel, Germany [25]). The F-test family (linear multiple regression analysis) was used with a type I error of 0.05 and a statistical power of 0.80 (type II error rate) for physical fitness components (i.e., static balance, muscle strength, power, coordination) as independent (predictor) variables. With references to a study by Moradi et al. [26] who included five predictor variables (e.g., muscle strength, muscular endurance, flexibility, speed, agility) and one dependent variable (either information processing speed or inhibitory control), we included four predictor variables and one dependent variable (composite score of attention) in our statistical model. Thus, a sample size of 53 participants would be needed to explore a medium to strong effect size of F^2^ = 0.25 [27] for our regression analysis. In the present study we are referring to test–retest reliability using intra class coefficients (ICC) for all physical fitness and cognitive tests which were assessed in our pilot study (pre-post testing of control group n = 22; [7]). The pilot study was conducted between August and November 2018 using a quasi-experimental study design (a 2-group repeated-measures design).

### Anthropometric data

Anthropometric data (body height, body mass, and BMI) was measured using standardized procedures [7]. Body mass index (BMI) was calculated using the standardized equation (mass/height [in kilograms per square meter]).

### Physical fitness

Static balance, muscle strength, power, and coordination were assessed in exercise rooms located within the kindergartens by specifically trained assessors. Every child was tested individually after performing one familiarization trial and after having received standardized verbal instructions and visual demonstration regarding the test procedures.

#### Single-leg stance test

Static balance was evaluated by using the single-leg stance test [28]. Children had to stand barefoot with eyes opened on the dominant leg which was assessed through the ball kick tests [29]. The stopwatch was started as soon as the nondominant leg was lifted in front with hip and knee joints both flexed at 90°. Children performed one trial up to a maximum of 30 s. If they were not able to pass 2 s in the first trial, they were asked to perform a second trial [30]. A child was considered as not capable of performing the single-leg stance test if he or she performed 2 unsuccessful trials. Time was measured by a stopwatch to the nearest one-tenth of a second and was stopped if the nondominant leg touched the floor or the child started hopping to achieve stability. The interrater reliability for the single-leg- stance test from our pilot study was ICC = 0.76 [7].

#### Standing long jump test

As a proxy of lower limbs muscle power, standing long jump performance was assessed. Children were instructed to jump with both feet starting from a parallel standing position as far as possible in horizontal direction aiming on landing on both feet [28]. The jumping distance from start to landing was taken using a measuring tape to the nearest 1.0 cm. A trial was considered as not valid if children lost balance during landing and fell backwards. Children performed 2 trials and the best trial was used for further analysis. In our pilot study the interrater reliability was ICC = 0.89 [7].

#### Handgrip strength test

Muscle strength was assessed using a handheld dynamometer (Jamar plus digital with LCD display). Therefore, children performed the handgrip strength test with the dominant hand which was assessed through reports of the kindergarten teachers [31] as the preferred hand when performing fine and gross motor tasks. Prior to the handgrip strength test, the hand’s span length of each participating child (diagonal length from tip of the little finger/pinky to the tip of the thumb) was assessed. According to the span length, we used level 1 (girls 14 cm; boys 10.8 cm) or level 2 (girls 14–19.1 cm; boys 10.8–20.1 cm) to enable an individualized biomechanical position for the handgrip strength test. The Jamar handheld dynamometer has 5 notches (levels) which can be adjusted depending on the individually hand span length. While sitting on a chair with shoulders relaxed and elbows flexed at 90°, the dynamometer had to be pressed continuously at maximum effort for at least 3–4 s [32]. The best of two trials was used for further analysis. Muscle strength was measured to the nearest 0.1 kg. In children aged 4–6 years, the handgrip strength test has proven to be reliable (ICC = 0.83; [7]).

#### Hopping on one leg test

For the assessment of coordination, the hopping on right/left leg test [33] was operationalized and performed alternately once with each leg. Children were instructed to hop on one leg as often as possible to a maximum of 20 hops. If takeoff and landing was achieved on the same foot and at least one time, a hop was considered valid. The interrater reliability in our pilot study was ICC = 0.60 for the right and ICC = 0.88 for the left leg, respectively [7]. For further analyzes, we computed a composite score as on overall measure of coordination by using the mean z-scores from each leg (right/left).

### Cognitive performance

Attention as one domain of cognition was assessed in quiet rooms in the respective kindergartens for each child individually by one specifically trained assessor.

#### Konzentrations-Handlungsverfahren für Vorschulkinder

We applied attention with the Konzentrations-Handlungsverfahren für Vorschulkinder [concentration-action procedure for preschoolers] (KHV-VK) [34]. Children had to sort 40 cards with familiar images as fast as possible but within a maximum time of 10 min in 4 different boxes. On every card, children had to find the key image (no, single, or double key images) in order to sort the card into the correct box. The KHV-VK measures and analyzes sorting time (working speed) as quantitative and error quote (working accuracy) as qualitative dimension of attention. The ICC in our pilot study were ICC = 0.43 for sorting time and ICC = 0.73 for correct cards [7]. Further, the test has been validated in children aged 4–6 years and proved to be sufficiently valid as a diagnostic procedure [34]. We calculated a composite score as an overall measure of attentional capacity using the mean of the z-scores of the individual dimensions of the KHV-VK (raw scores of sorting time as quantitative and error quote as qualitative dimension).

### Statistical analyses

Normality of data was assessed and confirmed using the Shapiro–Wilk test. Accordingly, descriptive statistics were reported as group mean values and standard deviations (SD). The relationship between measures of physical fitness and cognitive performance were tested using two-tailed Pearson correlation coefficients for continuous variables and Spearman rank correlation for nominal variables. According to Cohen [27], a correlation coefficient of r < 0.3 is considered weak, 0.3 ≤ r < 0.5 moderate, and r ≥ 0.5 strong. We defined age, sex, body height, body mass as covariates that may influence physical fitness and cognitive performance in children. Prior to the regression analyses, key assumptions were checked. One individual case was identified as outlier and excluded from further analyses as linear regression models are not robust towards outliers. All other key assumptions of our regression models were confirmed. Single linear regression models (unadjusted vs. adjusted for potential covariates) were calculated for attention (composite score and individual dimensions—dependent variable) and the composite score of physical fitness (independent variable). Subsequently, the relation between attention (composite score and individual dimensions—dependent variable) and the four measures of physical fitness (static balance, muscle strength, power, and coordination—independent variables) were analyzed by a stepwise multiple linear regression model to find physical fitness components that predict the variance of attention in early childhood. To ascertain if a predictor variable has a practically meaningful effect, we interpreted Cohen’s F for the single linear regression models and Cohen’s F^2^ for the multiple linear regression model. For Cohen’s F, we calculated the square root of (R^2^ divided by 1-R^2^) considering an effect as small = 0.10, medium = 0.25, or large = 0.40. For Cohen’s F^2^, R^2^ was divided by 1-R^2^ considering an effect as small = 0.02, medium = 0.15, or large = 0.35 [27]. The significance level was set at *p* < 0.05. As no performance differences were found between boys and girls, statistical analyses were computed using pooled data. All statistical analyses were performed using SPSS version 25.0 (IBM SPSS Statistics, Armonk, NY, USA).

## Results

Total descriptive characteristics of age, anthropometry, physical fitness, and cognitive performance are presented in Table 1. No injuries were reported during physical fitness testing. In terms of correlation analyses between covariates (age, sex, body height, body mass), physical fitness, and cognitive performance (data not shown), we found that standing long jump, hopping on one leg, and handgrip strength were related to age (*p* ≤ 0.05) showing medium to strong correlations coefficients (r = 0.32–0.59). Body height and body mass showed significant (*p* ≤ 0.05) medium to strong correlations (r = 0.27–0.66) with standing long jump, hopping on one leg, handgrip strength, and the qualitive dimension of the KHV-VK. Thus, age, body height, and body mass were included in the regression models as covariates. Additionally, all four measures of physical fitness were significantly (*p* ≤ 0.01) correlated with each other and with the composite score of attention (*p* ≤ 0.05).

**Table 1:** Descriptive characteristics

Variables	Girls *(n* = *30)*	Boys *(n* = *31)*	Total *(N* = *61)*	*p* (Cohen’s *d*)
	Mean ± *SD*	Mean ± *SD*	Mean ± *SD*	Between group differences
*Age and anthropometry*				
Age (years)	4.5 ± 0.6	4.5 ± 0.7	4.5 ± 0.6	0.769 (0.06)
Age (months)	57.9 ± 7.5	59.6 ± 7.2	58.7 ± 7.3	0.413 (0.21)
Body height (cm)	109.5 ± 6.3	112.0 ± 4.9	110.8 ± 5.7	0.089 (0.45)
Body mass (kg)	19.3 ± 3.2	19.2 ± 2.2	19.2 ± 2.7	0.874 (0.04)
BMI (kg/m^2^)	16.0 ± 1.5	15.2 ± 1.1	15.6 ± 1.4	0.033 (0.57)
*Physical fitness*				
Single-leg stance (max. 30 s)	13.6 ± 8.5	11.3 ± 8.0	12.5 ± 8.3	0.286 (0.29)
Standing long jump (cm)	77.8 ± 16.7	78.7 ± 22.4	78.3 ± 19.7	0.874 (0.04)
Hopping on right leg (max. 20)	14.3 ± 6.9	11.9 ± 7.7	13.2 ± 7.3	0.210 (0.33)
Hopping on left leg (max. 20)	12.7 ± 7.0	12.7 ± 7.5	12.7 ± 7.2	0.992 (0.01)
Composite score (coordination)	0.1 ± 0.9	− 0.1 ± 0.9	− 0.001 ± 0.9	0.454 (0.20)
Handgrip strength (kg)	7.1 ± 1.5	7.5 ± 1.9	7.3 ± 1.7	0.350 (0.25)
Composite score (physical fitness)*	0.02 ± 0.7	− 0.07 ± 0.9	− 0.02 ± 0.8	0.654 (0.11)
*Cognitive performance*				
Sorting time KHV-VK (in min)	7.2 ± 1.6	6.9 ± 2.0	7.1 ± 1.9	0.455 (0.20)
Correct cards KHV-VK (number)	30.6 ± 8.2	31.1 ± 6.8	30.9 ± 7.4	0.823 (0.06)
Composite score (attention)	− 0.1 ± 0.8	0.1 ± 0.7	0.00 ± 0.76	0.526 (0.17)

*BMI* body mass index

*Mean of the z-scores of each of the four physical fitness tests

In the single linear regression analyses (Table 2), the composite score of physical fitness was positively associated to the composite score of attention before and after adjusting for age, body height, and body mass (standardized ß = 0.40–0.43; *p* < 0.05). The effect size for the association between physical fitness and attention was considered small to medium in the adjusted model. The relationship between the composite score of physical fitness with the individual dimensions of attention (sorting time—quantitative; correct cards—qualitative) however, did not remain significant after adjustment.

**Table 2:** Single linear regression analyses using attention as dependent and a composite score of physical fitness as predictor variables

	Physical fitness (composite score)					
	R^2^	ß-Coeff	r^3^	95% CI	*p* Value	Effect size
*Attention (composite score)*						
Unadjusted model	0.183	0.43	0.43	0.172–0.609	**0.001**	**F = 0.47**
Adjusted model*	0.126**	0.40	0.34	0.091–0.630	**0.010**	**F**^**2**^** = 0.14**
*Attention (sorting time in min)*						
Unadjusted model	0.102	− 0.32	− 0.32	− 1.376– − 0.166	**0.013**	**F = 0.34**
Adjusted model*	0.067**	− 0.36	− 0.31	− 1.604– − 0.131	0.102	F^2^ = 0.07
*Attention (correct cards, number)*						
Unadjusted model	0.094	0.31	0.31	0.490–5.014	**0.018**	**F = 0.32**
Adjusted model*	0.069**	0.21	0.19	− 0.808–4.668	0.098	F^2^ = 0.07

Statistically significant (p < 0.05) relationships between physical fitness and attention are written in bold

*95% CI*, 95% confidence interval, *ß-Coeff.* standardized ß-coefficient, *r*^*3*^ partial correlation coefficient—association between dependent and predictor variables of regression analyses

*Adjusted for age, body height, body mass, **adjusted R^2^

The stepwise multiple linear regression analysis revealed a significant positive association between coordination (hopping on one leg) and the composite score of attention (standardized ß = 0.35; *p* < 0.01) showing a small to medium effect size (Table 3). Static balance, muscle strength, and power had not been included in the model as they were not significantly predicting attention in addition to coordination. Moreover, coordination also predicted the quantitative dimension of attention “sorting time” (standardized ß =  − 0.33; *p* < 0.05). No physical fitness component significantly predicted the qualitative dimension of attention “correct cards”.

**Table 3:** Stepwise multiple linear regression analysis using attention as dependent and physical fitness components as predictor variables

Variables	R^2^	ß-Coeff	r^3^	95% CI	*p* Value	Effect size
Attention (composite score) Coordination	0.106*	0.35	0.35	0.068–0.448	**0.009**	**F**^**2**^** = 0.12**
Attention (sorting time in min) Coordination	0.090*	− 0.33	− 0.33	− 1.239– − 0.141	**0.015**	**F**^**2**^** = 0.10**
Attention (correct cards, number) none	–	–	–	–	–	–

Statistically significant relationships (p < 0.05) between physical fitness and attention are written in bold

*95% CI* 95% confidence interval, *ß-Coeff.* standardized ß-coefficient, *r*^*3*^ partial correlation coefficient—association between dependent and predictor variables of regression analyses

*Adjusted R^2^; only those physical fitness components that predicted attention (composite score, quantitative and qualitative dimension) are noted here

## Discussion

Our findings indicate that physically fitter 4- to 6-year-old children (composite score of physical fitness) showed significantly better attentional capacity (composite score) than less fit children with a significant small-medium effect (F^2^ = 0.14). Additionally, our results illustrate that coordination as assessed by the hopping on one leg test had the strongest relation with the composite score and the quantitative dimension of attention. The physical fitness component coordination explained about 11% and 9% of the variance of attention with a weak to medium effect size.

Regarding the relation between physical fitness (composite score) and attention (single linear regression models) we cannot compare our results with other studies. There is no study available that analyzed the relationship between a total motor score and attention (composite score, individual dimensions of attention separately) in preschool children. Yet, there are studies reporting significant correlations between composite motor scores of physical fitness and domains of cognitive performance others than attention (e.g., visuospatial working) in preschoolers [8, 17].

In accordance with the study hypothesis, our results show that coordination is related to attention. As a proxy of coordination, we assessed the hopping on one leg test which is a complex and demanding exercise measuring dynamic balance, muscle strength, muscular endurance, bilateral body coordination, and coordination of rhythm. The neuromuscular demands during the execution are highly cognitively determined. While hopping, motor control and motor regulation are constantly needed to make up the performance in hopping (feedback-control mechanisms). Those mechanisms lead to co-activations between different parts of the central nervous system [35] which may stimulate motor performance, attention, and especially working speed (quantitative dimension of attention—sorting time), simultaneously and could explain the associations in our study. Our results are in accordance with the literature. Although, van der Fels et al. [11] did not specifically focus on attention, they found that fine motor skills, bilateral body coordination, and speed of movement (e.g., foot tapping, running in a zigzag) were significant associated with memory, visual processing, executive functions, and fluid intelligence in children. The authors assumed that the complex structure of physical exercises which were demanding and needed precise coordination during execution were stronger related to cognitive performance than simple and less complex exercises. Other studies support these findings by showing for instance that bilateral body coordination, the speed of movement (i.e., foot tapping) and agility had the strongest associations with fluid intelligence and attention in preschool children [10, 17, 36]. Thereby, higher levels of attentional capacity will prepare preschool children for school entry [22] and facilitate the transition from preschool to primary school. Additionally, academic achievements in primary school are related to attention at preschool age [24]. Given that the other fitness components (e.g., static balance, muscle strength, and power) could not improve the explained variance of the dependent variable (i.e., composite score and quantitative dimension of attention) they had not been included in the stepwise multiple regression model. These results are partly in line with the literature [8–10]. Niederer et al. [10] could not find an association of dynamic balance with attention nor for the relation between standing long jump and visuospatial working [8] or visual processing [9] or handgrip strength and visual processing [9]. The authors presumed that the tasks mentioned above were simpler to perform, less complex, and therefore required lower cognitive demands.

Nevertheless, the null results of the relation between static balance, muscle strength, power, and attention do not mean that those fitness components are not important for a child’s development during preschool age. Regardless of their relationship to one another (transfer effects), physical and cognitive development are capabilities which are highly “plastic” during early age [15, 37]. To maximize a child’s potential of a healthy and optimal development, physical fitness and cognitive performance have to be promoted and trained, whether separately or together. Niederer and colleagues [10] found out that future improvements of attentional capacity at school age was related to the former fitness level at preschool age which illustrates the close relationship between motor and cognitive development [17].

## Strength and limitations

The present study includes four different measures of physical fitness (i.e., static balance, muscle strength, power, and coordination) which were objectively assessed in children 4–6 years. We were focussing on attention and its dimensions, the quantitative (sorting time—working speed) and the qualitative (correct cards—working accuracy) dimension (attentional capacity) which we included in our statistical analyses separately. Further, we used two linear regression models to precisely analyze the relationship between physical fitness and attention. First, we calculated single linear regression analyses including possible covariates (i.e., age, body height, and body mass). Second, a stepwise multiple linear regression analysis was computed to find physical fitness components that predict the variance of attentional capacity in early childhood. Nonetheless, there are some limitations that have to be discussed. The cross-sectional design of our study neither allows cause-and-effect relationships nor an interpretation of direction of the association between physical fitness and attention. Furthermore, we included a relatively small none-representative sample size (N = 61). The participating children were selected from 3 kindergartens located in eastern Germany by convenience. Thus, more longitudinal studies with a representative sample under consideration of further covariates (e.g., socio-demographic, socio-economic background, parent’s attitude towards physical activity) are needed to examine the relationship of physical fitness and cognitive performance precisely and to detect the direction of association.

## Conclusion

The results of the present study indicate that higher performance in physical fitness is related to better attentional capacity already at preschool age. This association was mainly driven by a complex task of coordination (e.g., hopping on one leg) rather than by simple fitness tasks (e.g., static balance, muscle strength, and power). Educators, teachers, and parents should be aware of the close relationship between motor and cognitive development during preschool age [17]. Especially complex and joyful exercises requiring permanent motor control and motor regulation may positively interact with cognitive tasks [35, 38]. Nevertheless, more longitudinal and interventional research is needed to examine cause-effect relationships between physical fitness and attention at preschool age preferably using a larger randomly selected sample of preschoolers.

## Data Availability

The datasets used and analyzed during the current study are available from the corresponding author on reasonable request.

## Abbreviations

BMI: Body mass index
cm: Centimeter
ICC: Intra class coefficients
kg: Kilogram
KHV-VK: Konzentrations-Handlungsverfahren für Vorschulkinder
SD: Standard deviation
vs.: Versus
